# Small cell lung cancer with dermatomyositis: a case report

**DOI:** 10.3389/fonc.2024.1325991

**Published:** 2024-02-12

**Authors:** Xiaomin Guan, Shi Qiu, Yinghui Xu, Jianjiao Zu, Chao Sun, Ye Guo, Xu Wang, Kewei Ma

**Affiliations:** ^1^ Cancer Center, The First Hospital of Jilin University, Changchun, Jilin, China; ^2^ Dermatology Department, The First Hospital of Jilin University, Changchun, Jilin, China

**Keywords:** SCLC, small cell lung cancer, lung cancer, paraneoplastic syndrome, dermatomyositis

## Abstract

Dermatomyositis represents an autoimmune disorder characterized by notable skin and muscular manifestations. The annual incidence of dermatomyositis stands at approximately (5~10)/1 million individuals. Notably, patients with malignant tumors exhibit an elevated risk of developing dermatomyositis compared to the general population. However, in cases where dermatomyositis co-occurs with malignancy, the efficacy of hormone therapy alone tends to be suboptimal. Moreover, reports addressing the correlation between tumor treatment and the management of dermatomyositis are scarce. A 60-year-old male patient presented with dermatomyositis, initially manifesting through symptoms such as rash, muscle weakness, and dysphagia. Despite undergoing standard hormone therapy, there was no discernible improvement in the dermatomyositis symptoms. Considering the patient’s concomitant troublesome cough, further investigations were conducted, including CT, PET-CT, and pathological biopsy. These assessments confirmed the diagnosis of limited-stage small cell lung cancer (T1cN3M0 IIIB). Notably, in this patient, dermatomyositis was suspected to be a paraneoplastic syndrome associated with small cell lung cancer. Standard chemotherapy and radiotherapy were employed to treat the small cell lung cancer, resulting in partial remission after two treatment cycles. As the malignancy regressed, a notable improvement in dermatomyositis symptoms was observed, subsequently leading to a gradual reduction in the prescribed hormone dosage. In conclusion, we present a comprehensive case study of dermatomyositis as a paraneoplastic syndrome throughout the treatment process. The response to tumor therapy coincided with the amelioration of dermatomyositis symptoms. Therefore, diligent malignancy screening is imperative for patients diagnosed with dermatomyositis.

## Introduction

Dermatomyositis falls within the category of autoimmune myositis, which encompasses a group of rare autoimmune disorders distinguished by varying degrees of skin rashes and myopathy (1, 2). The precise etiology of dermatomyositis remains elusive, with factors such as viral infections, vascular conditions, environmental triggers, and both immune and non-immune-mediated mechanisms potentially contributing to its development (2). Notably, individuals diagnosed with dermatomyositis exhibit a significantly elevated risk of cancer compared to the general population (3). This heightened risk may be attributed to shared antigenic expressions between muscle and cancer cells (4). Dermatomyositis has been linked to a spectrum of malignancies, with a meta-analysis conducted by Olazagasti et al. revealing a notable increase in cancer risk, particularly for lymphatic and hematopoietic, lung, and ovarian cancers (3). Small cell lung cancer (SCLC), constituting 15%-20% of lung cancer cases, represents a highly aggressive neuroendocrine tumor with a pronounced proclivity for neuroendocrine activity. Consequently, it is frequently associated with various paraneoplastic syndromes, encompassing endocrine paraneoplastic syndromes (e.g., vasopressin hormone deficiency syndrome), neurological paraneoplastic syndromes (e.g., Lambert-Eaton syndrome), as well as hematologic and dermatologic syndromes (e.g., dermatomyositis) (5). Dermatomyositis, in this context, emerges as a relatively prevalent paraneoplastic syndrome.

The timely diagnosis and identification of the underlying cause of dermatomyositis hold paramount significance, given its status as a severe, yet treatable, multisystem disorder. Establishing a clear association between malignancy and dermatomyositis facilitates the early detection of cancer. The case we present provide comprehensive diagnostic and therapeutic timelines for both dermatomyositis and small cell lung cancer. For dermatomyositis, we have fully clarified the diagnosis from multiple aspects of clinical presentation, laboratory, physiology and pathology. We also dynamically monitor the patient before, during and after treatment for changes in dermatomyositis and lung cancer and give a clear comparison. This shows that the improvement of dermatomyositis symptoms and the shrinkage of lung cancer lesions are synchronized. Throughout the course of dermatomyositis treatment, our efforts sought to delve into the pathogenic mechanisms, ultimately culminating in a precise diagnosis. This case report further underscores the strong link between dermatomyositis and malignancy.

## Case presentation

**Figure 1:**
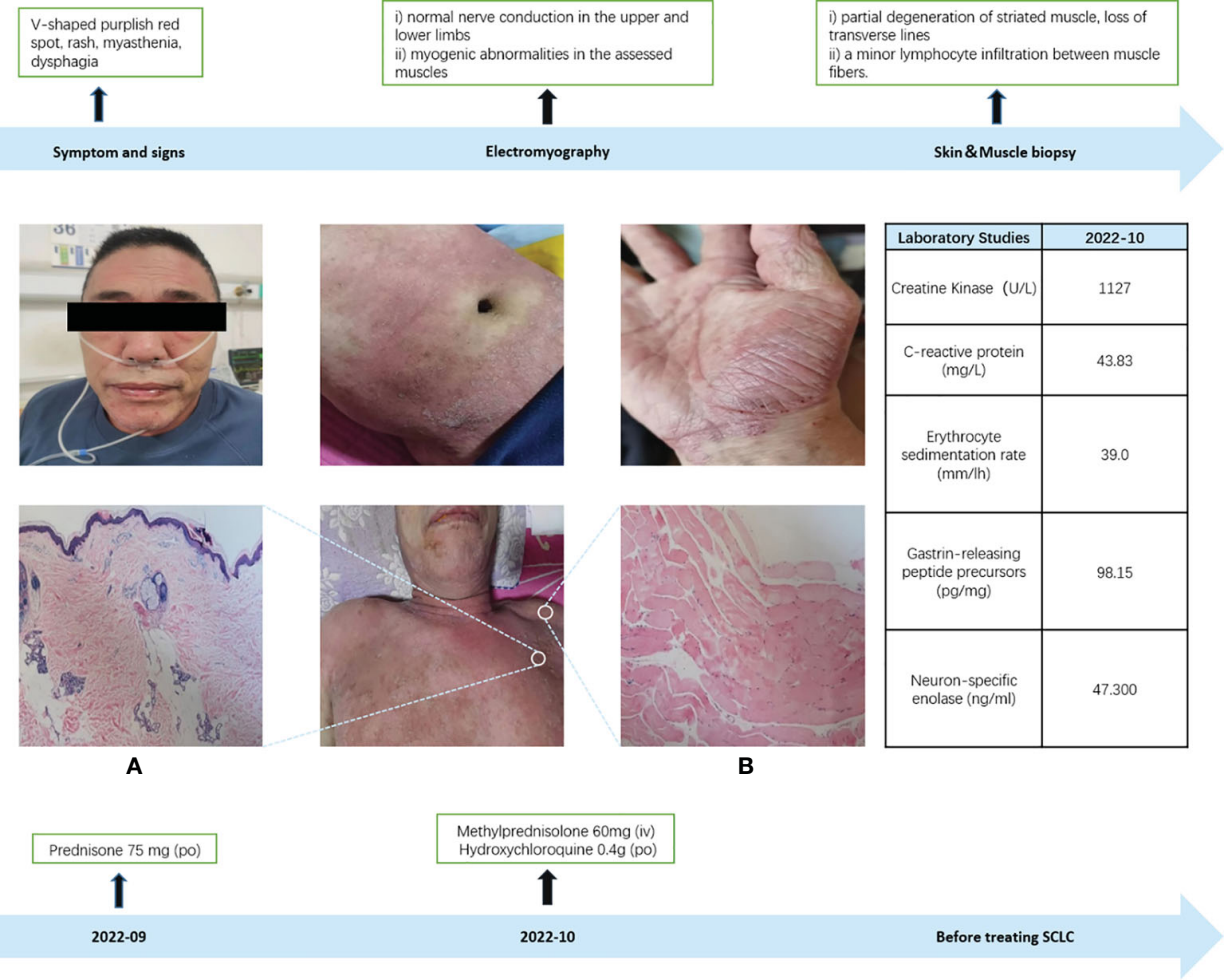
Dermatomyositis diagnosis. **(A)** Skin biopsy: The skin biopsy revealed marked hyperkeratosis with focal keratosis, thinning of the epidermis, increased pigment in the basal layer, dilation of superficial dermal blood vessels, a limited presence of lymphocytes in the vicinity, sporadic pigmented cells, and slight mucin deposition observed between the collagen fibers in the dermis (positive Acinlan staining), which did not exclude the possibility of dermatomyositis. **(B)** Muscle biopsy: In the muscle biopsy, there were evident signs of partial degeneration in striated muscle tissue, loss of transverse lines, and a minor infiltration of lymphocytes between muscle fibers.

In September 2022, a 60-year-old male patient with no history of chronic disease or chronic medication, no family history of cancer and a history of smoking was admitted to the hospital’s rheumatology and immunology department with complaints of facial and neck redness and swelling, which subsequently extended to the anterior and posterior chest regions. Over time, the patient also developed facial swelling, prominent purplish-red edema in and around the double eyelids and anterior chest area V, along with increasing weakness, bed confinement, and difficulty swallowing. Based on these clinical symptoms and signs, an initial diagnosis of dermatomyositis was established, and oral prednisone at a daily dose of 75mg was initiated (with reference to the standard treatment of 0.75-1mg·kg-1·d-1) (6). To further confirm the diagnosis of dermatomyositis, a battery of laboratory tests, electromyography, and a skin muscle biopsy were conducted. Routine laboratory investigations (complete blood count and biochemical profile) and the rheumatologic immunity investigations (ANA and ANCA) showed no abnormalities. However, on October 8, 2022, the creatine kinase level was measured at 1127U/L (normal range: 50-310U/L). Electromyography results indicated i) normal nerve conduction in the upper and lower limbs, and ii) myogenic abnormalities in the assessed muscles (**Appendix A**). The skin biopsy, while inconclusive for dermatomyositis, revealed certain characteristic features. Muscle biopsy results demonstrated partial degeneration of striated muscle, loss of transverse lines, and a minor lymphocyte infiltration between muscle fibers, thus firmly establishing the diagnosis of dermatomyositis (**Figure 1**). Subsequently, the treatment approach was adjusted to include intravenous methylprednisone at 60mg, supplemented by oral hydroxychloroquine at 0.4g. On October 11, 2022, laboratory assessments showed a creatine kinase level of 639U/L. However, despite these interventions, there was no significant improvement in the patient’s clinical presentation.

**Figure 2:**
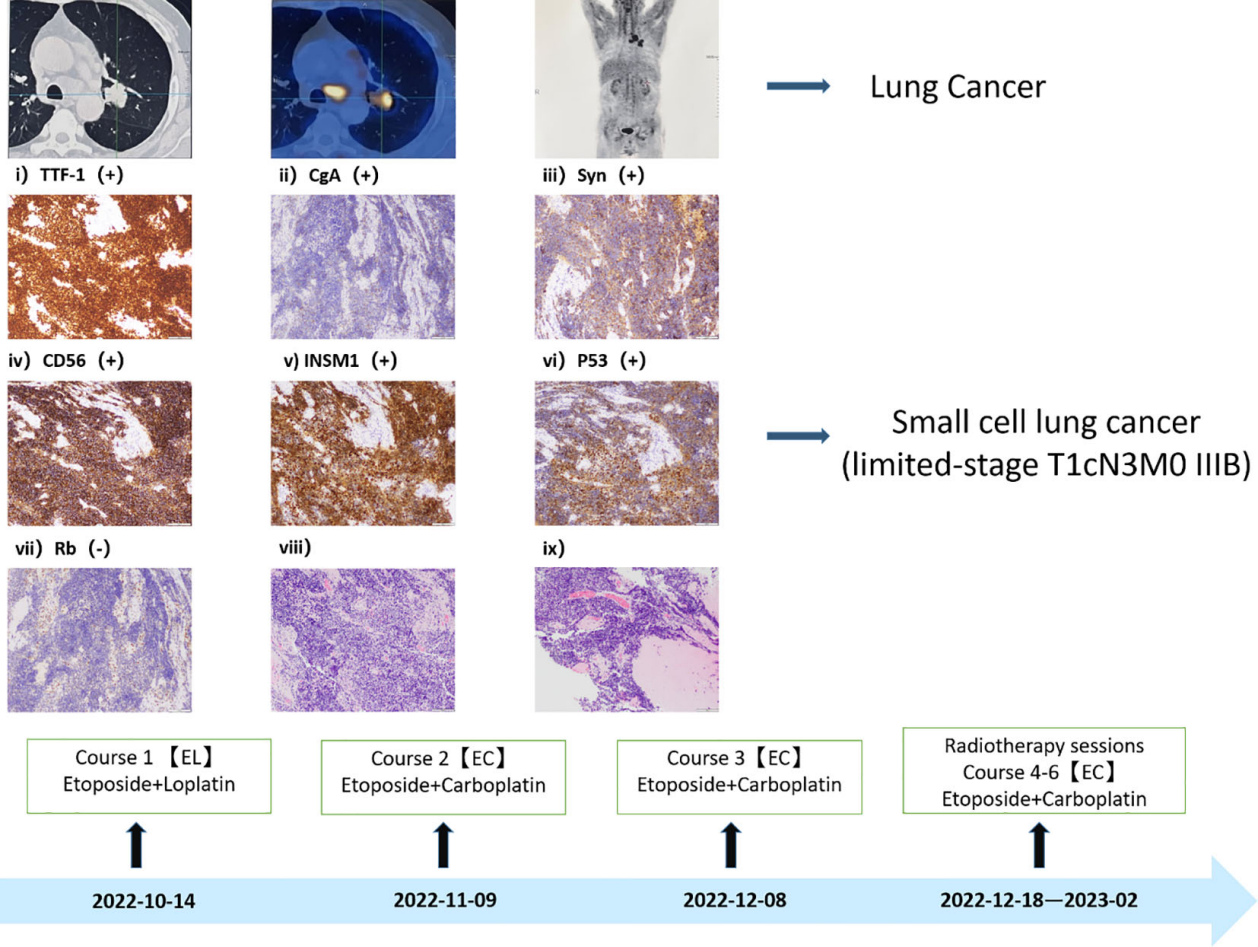
Small cell lung cancer diagnosis. The diagnosis of Immunohistochemistry: CK (AE1/AE3) (+)、TTF-1(+)、P40(-)、CgA (+) 、Syn (+) 、CD56 (+) 、INSM1 (+) 、Ki67 (positivity rate 80%) 、P53 (+) 、Rb (-) 、LCA (-) 、CK7 (A small amount of +).

Given the recent emergence of an irritating dry cough in the patient, unaccompanied by hemoptysis, we opted to conduct CT and PET-CT examinations. The findings revealed a space-occupying lesion in the left lung, characterized by an escalating nodular density and increased glucose metabolism at the posterior extremity of the upper lobe tip of the left lung. This prompted the consideration of a central lung mass, along with the identification of multiple intramediastinal and left hilar lymph node metastases. To conclusively establish the nature of the lesion, a bronchoscopic biopsy was performed, confirming the diagnosis of small cell lung cancer (limited-stage, T1cN3M0 IIIB) (**Figure 2**). With this confirmation, our suspicion that lung cancer might underlie the patient’s dermatomyositis gained credence, leading us to initiate a dual treatment approach addressing both conditions. Taking into account the patient’s pathological subtype, disease stage, and overall physical condition, we formulated a treatment plan involving a combination of chemotherapy and radiotherapy (The specific plan was as follows: for the first course of treatment, etoposide combined with loplatin; the second to sixth courses were etoposide combined with carboplatin) (7). Consequently, the patient commenced the initial chemotherapy regimen, comprising etoposide and cisplatin, on October 14, 2022.

On October 17, 2022, following the initial chemotherapy session, the patient’s creatine kinase levels were reevaluated, yielding a reading of 166U/L (within the normal range of 50-310U/L). Subsequently, creatine kinase levels and lung CT scans were reassessed after the second chemotherapy cycle. The patient exhibited a creatine kinase level of 78U/L, accompanied by a reduction in the lung mass to 1.1cm in size. Following the third chemotherapy session, the patient underwent radiation therapy. Over the subsequent months, until February 2023, the patient completed three additional rounds of chemotherapy and was subjected to a reevaluation following a cumulative total of six chemotherapy cycles and one radiation therapy session. The creatine kinase level was measured at 33U/L, and further shrinkage of the mass was observed. Concurrently, as hormone dosages were gradually tapered, the patient experienced an amelioration of symptoms, including muscle weakness, dysphagia, and skin rashes. By March 2023, the patient required only a daily oral dose of 10mg of Prednisone for maintenance.

**Figure 3:**
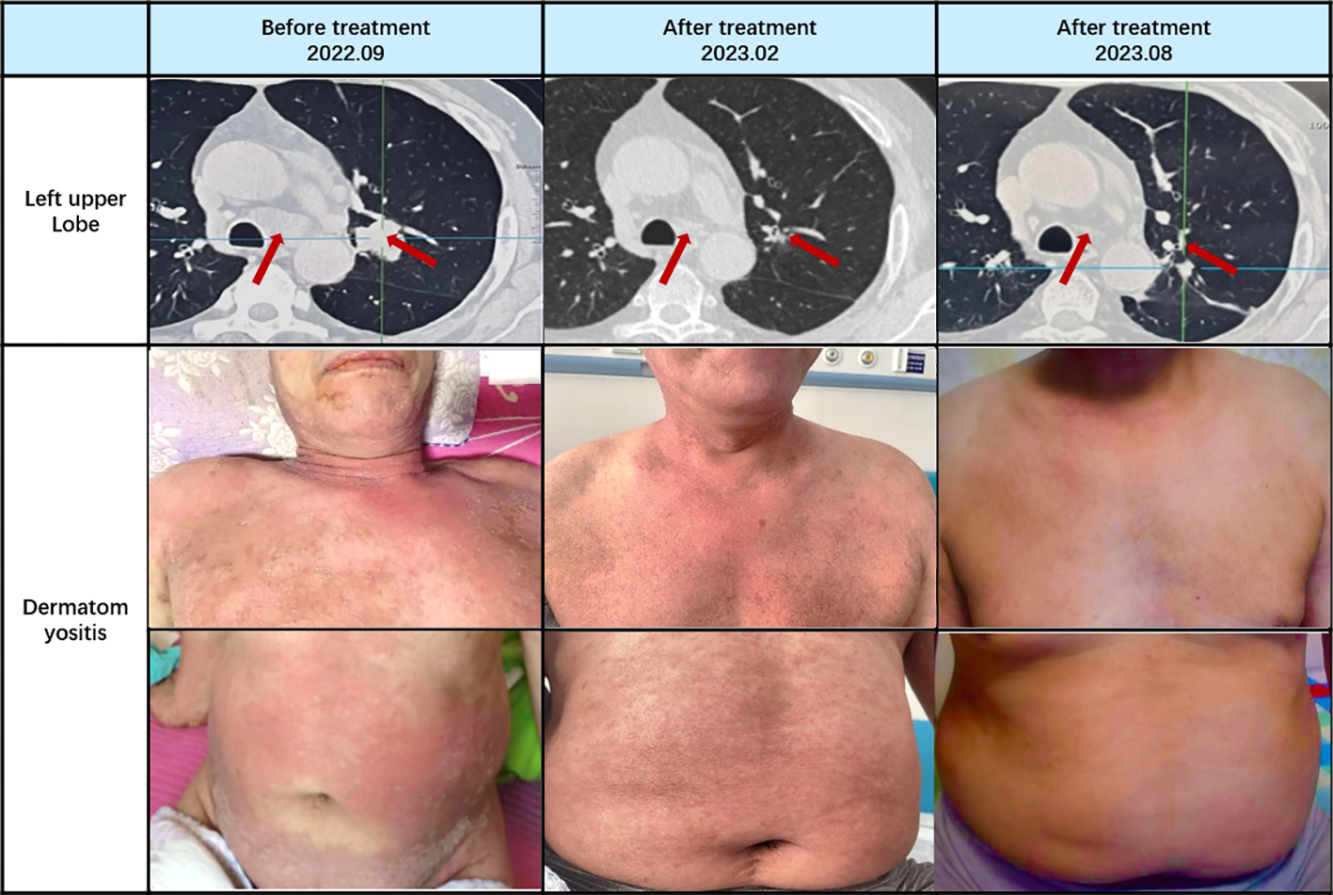
Therapeutic outcomes in small cell lung cancer (SCLC) and dermatomyositis.

**Figure 4:**
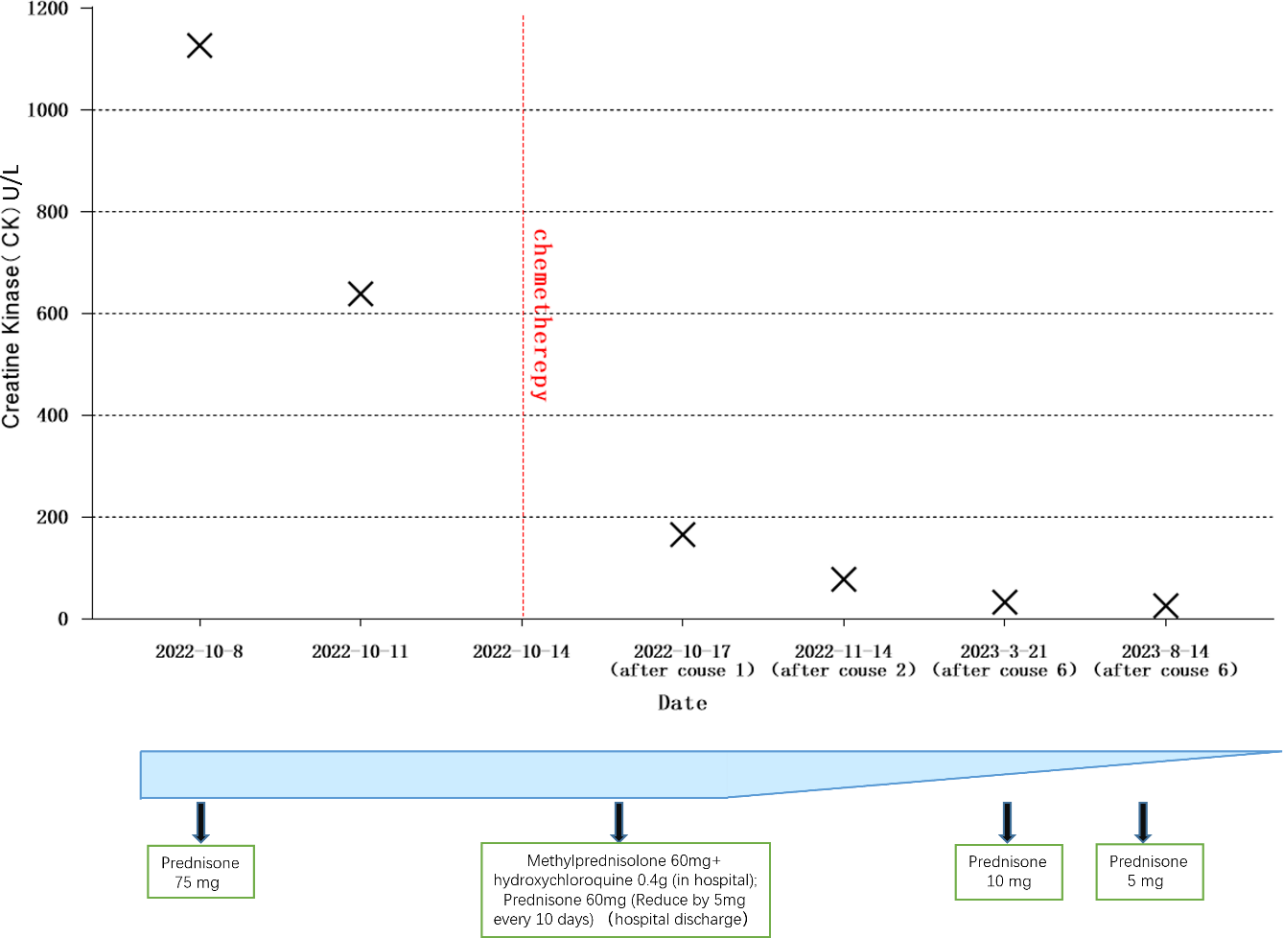
Creatine kinase changes and hormone dosage.

Continued patient follow-up until August 2023 revealed a significant reduction in the primary lung lesion, accompanied by the gradual resolution of dermatomyositis-related symptoms, such as skin rashes. Laboratory assessments recorded a creatine kinase level of 26U/L. Moreover, the oral hormone dosage was further reduced to 5mg of prednisone (**Figures 3**, **4**).

## Discussion and conclusion

Dermatomyositis is a rare condition, with an approximate incidence ratio of 2:1 in both genders and two distinct age peaks observed between the ages of 5 and 15 years and 50 and 60 years (8). However, recent research conducted by Walid Shalata et al. in 2022 reported similar gender ratios (9), while another study by Fujita et al., analyzing dermatomyositis-associated cancer patients from the period spanning from 1947 to 2000, contradicted these statistics by revealing that male patients accounted for approximately 76.2% of the cases (10). This inconsistency may be attributed to limited representation of patient cohorts failing to accurately reflect broader population dynamics. Furthermore, although adolescent dermatomyositis is frequently documented in literature, its co-occurrence with lung cancer remains infrequent (11).

The precise pathogenesis of dermatomyositis remains incompletely understood. However, its characteristics, including tissue inflammation, associated vasculitis, frequent autoantibody presence, evidence of T-cell-mediated myotoxicity, or complement-mediated microangiopathy, its frequent co-occurrence with other autoimmune disorders, and its positive response to immunotherapy, collectively suggest an autoimmune nature (12). Aberrant immune function within the body may contribute to the development of dermatomyositis. As an autoimmune ailment, dermatomyositis can augment the risk of cancer due to its potential to incite chronic inflammation.

When dermatomyositis manifests as a paraneoplastic syndrome, it is termed “tumor-associated dermatomyositis.” Molecular mimicry plays a pivotal role in the context of tumor-associated dermatomyositis. Tumor cells possess immunogenic properties, and the presentation of abnormal antigens secreted by these neoplasms can stimulate B cell activation and subsequent antibody production. These abnormal antibodies, owing to structural similarities with tumor antigens present in skin or muscle tissues, can erroneously target normal components, thereby inducing tissue damage upon recognition. This process involves the activation of C3, resulting in the formation of C3b, C3bNEO, C4b fragments, as well as membrane lysis attack complexes (MAC). Consequently, C3b, C4b, and MAC accumulate on the capillary walls, leading to endothelial cell swelling and vacuolization. Simultaneously, the activation of complement-related cytokines and chemokines ensues, upregulating vascular cell adhesion molecules (VCAM-1) and intercellular adhesion molecules (ICAM-1) on endothelial cells. T cells and macrophages, by binding to these adhesion molecules through their surface integrin, traverse endothelial cells and infiltrate muscle tissue, releasing various proinflammatory factors, including TNF-α, thereby fostering tissue damage. Consequently, in tissue pathology, we commonly observe the infiltration of inflammatory cells surrounding capillaries, capillary necrosis, and muscle bundle rupture or atrophy (4, 12–14). In summary, cancer can trigger autoimmune diseases via cross-immune reactions between antibodies and structurally analogous antigenic components found in muscle and skin tissues.

The presence of malignancy within 8 years of an inflammatory myopathy diagnosis, including occurrences during or after myositis episodes, raises the possibility of cancer-associated myositis (CAM). Recent population-based studies provide substantial evidence that the majority of malignancies manifest concurrently with the onset of myopathy. The temporal alignment between tumor treatment and the resolution of muscle symptoms supports the inference that CAM may indeed represent a paraneoplastic syndrome (15). Patients with uncomplicated dermatomyositis and those diagnosed with dermatomyositis associated with malignancies exhibit notable distinctions in terms of peak age of onset, likelihood of presenting characteristic symptoms, and laboratory findings. Retrospective analyses indicate that older individuals with dermatomyositis are at a heightened risk of malignancy (15, 16). Manifestations of dermatomyositis, particularly those involving the muscular and cutaneous systems, demonstrate a stronger association with malignant neoplasms. Patients with severe distal muscle weakness, respiratory muscle involvement leading to respiratory insufficiency, and dysphagia are at an elevated risk of cancer. In muscle biopsies from dermatomyositis patients, the presence of infiltrating inflammatory cells in the absence of perifascicular atrophy can potentially serve as a biomarker for an increased cancer risk. Furthermore, dermatomyositis-related skin manifestations such as leukocytic vasculitis, skin necrosis, periungual erythema, ulceration, and the characteristic V-sign are all indicative of potential malignancy (17). Some researchers argue that patients with dermatomyositis complicated by malignancies exhibit elevated erythrocyte sedimentation rates and C-reactive protein levels, along with reduced complement C4 levels, whereas individuals with uncomplicated dermatomyositis tend to have higher levels of creatine kinase and lactate dehydrogenase (17–19).

Based on the diagnostic criteria for dermatomyositis as proposed by Bohan and Peter in 1975, the following criteria apply: i) myasthenia; ii) confirmed myositis via muscle biopsy; iii) elevated serum creatine kinase levels; iv) abnormal electromyography; v) presence of characteristic skin lesions (20). Our patient unequivocally meets the diagnostic criteria for dermatomyositis. Standard hormone therapy failed to yield improvement in the patient’s dermatomyositis and, in fact, resulted in deterioration. Coupled with the patient’s persistent irritating cough, sputum production, pre-existing dysphagia, and abnormal laboratory findings, a comprehensive evaluation was initiated, encompassing CT and PET-CT scans. Consequently, a lung space-occupying lesion was identified, with pathological biopsy confirming its nature as small cell lung cancer. Subsequently, a standardized treatment approach involving chemotherapy for lung cancer and concurrent hormone therapy for dermatomyositis was instituted. Following two cycles of chemotherapy, CT scans revealed a reduction in the patient’s lung tumor size from 2.87 * 2.46cm to 1.1cm, concomitant with a decrease in creatine kinase levels from 1127U/L to 78U/L. After the completion of six chemotherapy cycles and one round of radiotherapy, further shrinkage of the lung tumor occurred, accompanied by an amelioration of dermatomyositis symptoms. These outcomes underscore the synchronous therapeutic effects in the management of both lung cancer and dermatomyositis.

Currently, the patient remains free from lung tumor recurrence, with no discernible dermatomyositis symptoms. It is worth noting that approximately 30% of dermatomyositis cases are associated with malignant tumors (21). In cases where hormone therapy proves ineffective for dermatomyositis patients, a meticulous examination of their medical history and symptomatology is imperative to discern potential etiological factors, facilitating the early detection of malignant tumors and the prompt initiation of effective therapeutic measures.

## Data availability statement

All data generated or analyzed during this study are included in this published article. Data sharing is not applicable to this article as no datasets were generated or analyzed during the current study.

## Ethics statement

Written informed consent was obtained from the patients/participants for the publication of this case report.

## Author contributions

XG: Data curation, Writing – original draft, Writing – review & editing. SQ: Data curation, Formal analysis, Writing – review & editing. YX: Formal analysis, Writing – review & editing. JZ: Formal analysis, Writing – review & editing. CS: Formal analysis, Writing – original draft. YG: Formal analysis, Writing – review & editing. XW: Conceptualization, Formal analysis, Writing – review & editing. KM: Conceptualization, Writing – review & editing.
