# Supplementary material for: Small cell lung cancer with dermatomyositis: a case report
Source: Front Oncol. 2024 Feb 12;14:1325991. doi: 10.3389/fonc.2024.1325991 (PMC10898245; doi:10.3389/fonc.2024.1325991)
Supplement: Supplementary file 1 [file DataSheet_1.docx]

Appendix A: Electromyography.

Conclusion: i) normal nerve conduction in the upper and lower limbs; ii) myogenic abnormalities in the assessed muscles

| ***Motion conduction velocity*** | ***latentperiod*** | ***amplitude*** | ***distance*** | ***Conduction velocity*** | ***area*** | ***Mean F-M latentperiod*** | ***Minimum F latentperiod*** |
| --- | --- | --- | --- | --- | --- | --- | --- |
|  | ***ms*** | ***mv*** | ***mm*** | ***m/s*** | ***ms*mv*** | ***ms*** | ***ms*** |
| **Ulnar nerve movement right** | | | | | | | |
| Wrist - ADM | 2.69 | 9.7 |  |  | 26.9 | 27.4 | 29.7 |
| Under the elbow - Wrist | 6.52 | 9.4 | 200 | 52.2 | 25.5 |  |  |
| Above the elbow - Under the elbow | 8.58 | 9.1 | 110 | 53.4 | 23.4 |  |  |
| **Median nerve movement right** | | | | | | | |
| Wrist - APB | 3.21 | 6.8 |  |  | 19.1 | 25.8 | 27.6 |
| Elbow - Wrist | 7.63 | 6.1 | 245 | 55.4 | 17.0 |  |  |
| **Tibial nerve movement right** | | | | | | | |
| Ankle - AH | 3.29 | 10.8 |  |  | 18.7 | 44.4 | 46.9 |
| Popliteal fossa - Ankle | 12.4 | 4.8 | 410 | 45.0 | 14.5 |  |  |
| **Common peroneal nerve movement right** | | | | | | | |
| Ankle - EDB | 2.88 | 4.1 |  |  | 14.0 |  |  |
| Under the knee - Ankle | 10.1 | 3.6 | 310 | 42.9 | 12.3 |  |  |
| Above the knee - Under the knee | 12.4 | 3.4 | 100 | 43.5 | 11.7 |  |  |
| ***Sensory conduction velocity*** | ***latentperiod*** | ***amplitude*** | ***area*** | ***distance*** | ***Conduction velocity*** |  |  |
|  | ***ms*** | ***uv*** | ***ms*uv*** | ***mm*** | ***m/s*** |  |  |
| **Ulnar nerve sensation right** | | | | | | | |
| Finger V - Wrist | 2.66 | 13.1 |  | 140 | 52.6 |  |  |
| **Median nerve sensation right** | | | | | | | |
| Finger II - Wrist | 3.06 | 16.7 |  | 165 | 53.9 |  |  |
| Finger III - Wrist | 2.99 | 13.4 |  | 175 | 58.5 |  |  |
| **Superficial peroneal nerve sensation right** | | | | | | | |
| Ankle - Instep | 2.46 | 18.8 |  | 125 | 50.8 |  |  |
| **Sural nerve sensation right** | | | | | |  |  |
| Mid-calf - Lateral malleolus | 2.15 | 12.4 |  | 120 | 55.8 |  |  |
|  |  |  |  |  |  |  |  |
| ***Data of EMG MUP*** | ***MUP*** | |  |  |  |  |  |
|  | ***Time limit*** | ***amplitude*** |  |  |  |  |  |
| Right Deltoid muscle | 8.3 | 693 |  |  |  |  |  |
| Right Medial head of the quadriceps | 10.6 | 759 |  |  |  |  |  |
| Right Biceps | 9.0 | 534 |  |  |  |  |  |
| Left Tibialis anterior | 12.8 | 793 |  |  |  |  |  |
| Right Tibialis anterior | 13.2 | 1432 |  |  |  |  |  |
|  |  |  |  |  |  |  |  |
| ***Muscle*** | ***Insert act*** | ***Spontaneous*** | | | | ***Recruitment*** |  |
|  | ***-*** | ***Fib*** | ***Positive*** | ***Fasc*** | ***CRD*** | ***IP*** |  |
| Right Deltoid muscle | Normal | - | - | - | None | Early Recruitment |  |
| Right Biceps | Normal | - | - | - | None | Mixture |  |
| Right Medial head of the quadriceps | Normal | - | - | - | None | Interference |  |
| Right Tibialis anterior | Normal | - | - | - | None | Interference |  |
| Left Tibialis anterior | Normal | - | - | - | None | Interference |  |
